# Genetic Control of Maize Shoot Apical Meristem Architecture

**DOI:** 10.1534/g3.114.011940

**Published:** 2014-05-22

**Authors:** Addie M. Thompson, James Crants, Patrick S. Schnable, Jianming Yu, Marja C. P. Timmermans, Nathan M. Springer, Michael J. Scanlon, Gary J. Muehlbauer

**Affiliations:** * Department of Agronomy and Plant Genetics, University of Minnesota, St. Paul, Minnesota 55108; † Department of Genetics, Development and Cell Biology, and Agronomy, Iowa State University, Ames, Iowa 50011; ‡ Department of Agronomy, Iowa State University, Ames, Iowa 50011; § Cold Spring Harbor Laboratory, Cold Spring Harbor, New York 11724; ** Department of Plant Biology, University of Minnesota, Saint Paul, Minnesota 55108; †† Department of Plant Biology, Cornell University, Ithaca, New York 14853

**Keywords:** shoot apical meristem, plant morphology, QTL, maize development, IBMRIL

## Abstract

The shoot apical meristem contains a pool of undifferentiated stem cells and generates all above-ground organs of the plant. During vegetative growth, cells differentiate from the meristem to initiate leaves while the pool of meristematic cells is preserved; this balance is determined in part by genetic regulatory mechanisms. To assess vegetative meristem growth and genetic control in *Zea mays*, we investigated its morphology at multiple time points and identified three stages of growth. We measured meristem height, width, plastochron internode length, and associated traits from 86 individuals of the intermated B73 × Mo17 recombinant inbred line population. For meristem height-related traits, the parents exhibited markedly different phenotypes, with B73 being very tall, Mo17 short, and the population distributed between. In the outer cell layer, differences appeared to be related to number of cells rather than cell size. In contrast, B73 and Mo17 were similar in meristem width traits and plastochron internode length, with transgressive segregation in the population. Multiple loci (6−9 for each trait) were mapped, indicating meristem architecture is controlled by many regions; none of these coincided with previously described mutants impacting meristem development. Major loci for height and width explaining 16% and 19% of the variation were identified on chromosomes 5 and 8, respectively. Significant loci for related traits frequently coincided, whereas those for unrelated traits did not overlap. With the use of three near-isogenic lines, a locus explaining 16% of the parental variation in meristem height was validated. Published expression data were leveraged to identify candidate genes in significant regions.

Differences in plant morphology in part reflect differences in organ shape, number, and size. Mutant analysis has been an excellent tool to dissect the major regulators controlling plant morphology. In addition, quantitative trait locus (QTL) mapping has been conducted in many plant species and on numerous traits impacting plant morphology, *e.g.*, leaf or leafy head size and shape (Jiang *et al.* 2000; Tian *et al.* 2011; Jun *et al.* 2013; Yu *et al.* 2013), root architecture (Johnson *et al.* 2000; Loudet *et al.* 2005; Courtois *et al.* 2009; Hochholdinger and Tuberosa 2009), fruit size and shape (Grandillo *et al.* 1999; Frary *et al.* 2000; Causse *et al.* 2004), and whole-plant or inflorescence architecture (Clark *et al.* 2006; Upadyayula *et al.* 2006; Lauter *et al.* 2008). Most QTL mapping studies have focused on traits that were measured on mature organs, although some have targeted features of the maize embryo (Yang *et al.* 2012; Moore *et al.* 2013). An understanding of the genetic control of the morphology of undifferentiated tissue types may lead to insight into the morphology and development of differentiated tissue types.

The shoot apical meristem (SAM) contains a set of undifferentiated stem cells and forms a vital control center for plant growth and development. It produces all aerial organs of the plant, including lateral shoots, leaves, and flowers and, together with environmental cues, determines plant architecture (Wang and Li 2008). The morphology of the SAM is constrained by the balance between organogenesis and stem cell maintenance. Without this balance, the meristem either depletes its supply of stem cells during leaf formation, leading to developmental arrest, or overproliferates stem cells and fails to initiate leaves (Barton 2010). In maize, the SAM is initiated during the transition stage of embryogenesis, and its dome-like structure begins to form around the coleoptilar stage. The central zone of the meristem contains the stem cells, whereas organogenesis takes place in the peripheral zone. Based on mutant analysis and transcript profiling, the function of organogenesis takes place before meristem maintenance begins in maize (Vollbrecht *et al.* 2000; Takacs *et al.* 2012). Leaves are formed from the SAM via recruitment of 100−200 leaf initials termed founder cells (Poethig 1984). About five leaves develop in the maize embryo between fertilization and seed maturation and quiescence; leaf development and growth resumes upon germination. Leaf primordia are initiated at regular intervals; the time between leaf initiation is measured in plastochrons (P) (Sharman 1942b; Sylvester *et al.* 1990). The importance of the SAM for growth and development has led to numerous genetic studies in which SAM function is investigated. Most of these studies focused on mutant analysis and uncovered several regulatory processes acting in the SAM. In some cases these mutants resulted in alteration of SAM size and/or shape as well as whole-plant morphology.

In *Arabidopsis*, a negative feedback loop between CLAVATA (CLV) and the homeobox gene *WUSHEL* (*WUS*) is a primary regulator of stem cell number and thereby SAM size (Schoof *et al.* 2000; Wang and Li 2008). Defects in CLV receptor-ligand signaling lead to enlarged meristems (Leyser and Furner 1992; Clark *et al.* 1993, 1995; Kayes and Clark 1998), whereas plants defective in *WUS* show impaired meristem maintenance (Laux *et al.* 1996). Maize mutants in this pathway include *faciated ear2* (*fea2*) (Taguchi-Shiobara *et al.* 2001) and *compact plant2* (*ct2*) (Bommert *et al.* 2013), which initially were identified based on their inflorescence phenotype but also affect the size of the vegetative meristem. Another maize mutant, *thick tassel dwarf* (*td1*) (Taguchi-Shiobara *et al.* 2001), is similar in function to CLV1 in inflorescence and floral meristems but in the vegetative SAM is more akin to the BAM genes (DeYoung *et al.* 2006), which have multiple functions throughout development (Lunde and Hake 2009).

Another major category of genes shown to affect meristem size is the *Knotted-1-like homeobox* (*KNOX*) genes. Maize plants without functional *Knotted-1* (*Kn1*), the founding member of this gene family (Hake *et al.* 1989; Vollbrecht *et al.* 1991), are unable to maintain the shoot meristem (Kerstetter *et al.* 1997; Vollbrecht *et al.* 2000). In maize, kn1 mutants display a decrease in meristem size with penetrance dependent on genetic background, providing a clear indication for the presence of natural variation in pathway regulating SAM activity (Vollbrecht *et al.* 2000). Meristem termination phenotypes also were observed in orthologs of kn1, *Arabidopsis STM* (Long *et al.* 1996) and rice *OSH1* (Tsuda *et al.* 2011). Maize *KNOX* genes, such as *rough sheath1*, *gnarley1/KNOX4*, and *liguleless3* (*lg3*) and *lg4* (Schneeberger *et al.* 1995; Foster *et al.* 1999; Muehlbauer *et al.* 1999; Bauer *et al.* 2004), are expressed specifically in the SAM, but possibly due to redundancy, these mutants are not known to have an effect on SAM size (Hake *et al.* 2004; Bolduc *et al.* 2014).

KNOX proteins act by controlling the ratio of plant hormones in the SAM to maintain meristematic identity (Kyozuka 2007). Indeed, the regulation and patterning of many plant hormones is vital to the function of the meristem (see Hay *et al.* 2004 and Vanstraelen and Benková 2012 for reviews). Cytokinin promotes cell division, whereas auxin promotes organogenesis in the peripheral zone of the meristem (Pernisová *et al.* 2009). Perturbation of cytokinin biosynthesis leads to loss of the SAM (Yanai *et al.* 2005), whereas mutations in cytokinin response regulators, such as the maize mutant *aberrant phyllotaxy1* (*abph1)* cause larger meristems to form (Jackson and Hake 1999; Giulini *et al.* 2004). Without auxin transport the SAM loses its ability to form organs (Reinhardt *et al.* 2000; see Gallavotti 2013 and Forestan and Varotto 2012 for reviews). Gibberellins and brassinosteroids interact with auxin and cytokinin pathways to regulate their ratio during plant development (Vanstraelen and Benková 2012).

SAM establishment and function also are impacted by the activity of small RNAs (Zhang *et al.* 2006; see Axtell 2013 for microRNA review). During SAM formation in *Arabidopsis*, microRNA394 (miR394) moves from the protoderm to the underlying cell layers to define stem cell location (Knauer *et al.* 2013). In addition, the correct spatiotemporal regulation of class III homeodomain leucine zipper (HD-ZIPIII) transcription factors by miR166 is essential for normal meristem function, As such, key regulators in miRNA biogenesis or function show meristem defects when affected, *e.g.*, *ago1* (Vaucheret *et al.* 2004), *ago10* (Liu *et al.* 2009), and *dcl1* (Schauer *et al.* 2002). Likewise, maize *leafbladeless* and *ragged seedling2*, which encode essential components in the biogenesis of *trans*-acting small interfering RNAs, regulate meristem function through their effect on auxin response as well as the expression domain of miR166 and HD-ZIPIII transcription factors (Nogueira *et al.* 2007; Douglas *et al.* 2010). Mutants in this pathway in rice, *sho* mutants, display variable morphological differences in the SAM, the shape of which correlates with variation in phyllotaxy and plastochron timing (Itoh *et al.* 2000; Nagasaki *et al.* 2007), linking SAM architecture to plant morphology.

Chromatin structure and remodeling regulators are parts of yet another process linked to stem cell maintenance (Shen and Xu 2009; for review see Wagner 2003; Kwon and Wagner 2007; Sang *et al.* 2009). Chromatin remodeling pathway components also interact with the cytokinin response pathway (Efroni *et al.* 2013), leading to cross-talk among the regulatory processes.

Besides these regulatory mechanisms that ultimately effect gene expression, several recent studies are revealing the downstream processes that impact SAM function. These include enzymes that vary the properties of the cell wall, as well as metabolic processes (Peaucelle *et al.* 2011; Kierskowski *et al.* 2012). For instance, maize *bladekiller1-R* (*blk1-R*), a thiamine auxotroph, is defective in both meristem maintenance and organ initiation and displays progressively decreasing SAM size (Woodward *et al.* 2010).

These processes and pathways act and interact to form a complex regulatory network controlling SAM morphogenesis and maintenance. Although many aspects of the maize SAM have been studied through mutant and expression analysis, an understanding of the genetic control of natural variation in SAM architecture is unknown.

The objectives of this study were to examine SAM morphology during vegetative development, calculate heritability and segregation of SAM measurements in a recombinant inbred line (RIL) population map QTL to determine the genetic architecture controlling natural variation in SAM morphology, and to use expression data to identify candidate genes involved in regulating and responding to changes in meristem architecture.

## Materials and Methods

### Plant materials

Two sets of plant materials were used in this study: 86 lines from the intermated B73 × Mo17 recombinant inbred line (IBMRIL) population and the B73 and Mo17 parents (Supporting Information, Table S1; Lee *et al.* 2002); and a set of three near-isogenic lines (NILs) selected from the 150 B73 × Mo17 NIL population (Table S1; Eichten *et al.* 2011). These NILs were backcrossed three times and self-pollinated for four to six generations.

### Plant growth and experimental design

**Figure 1:**
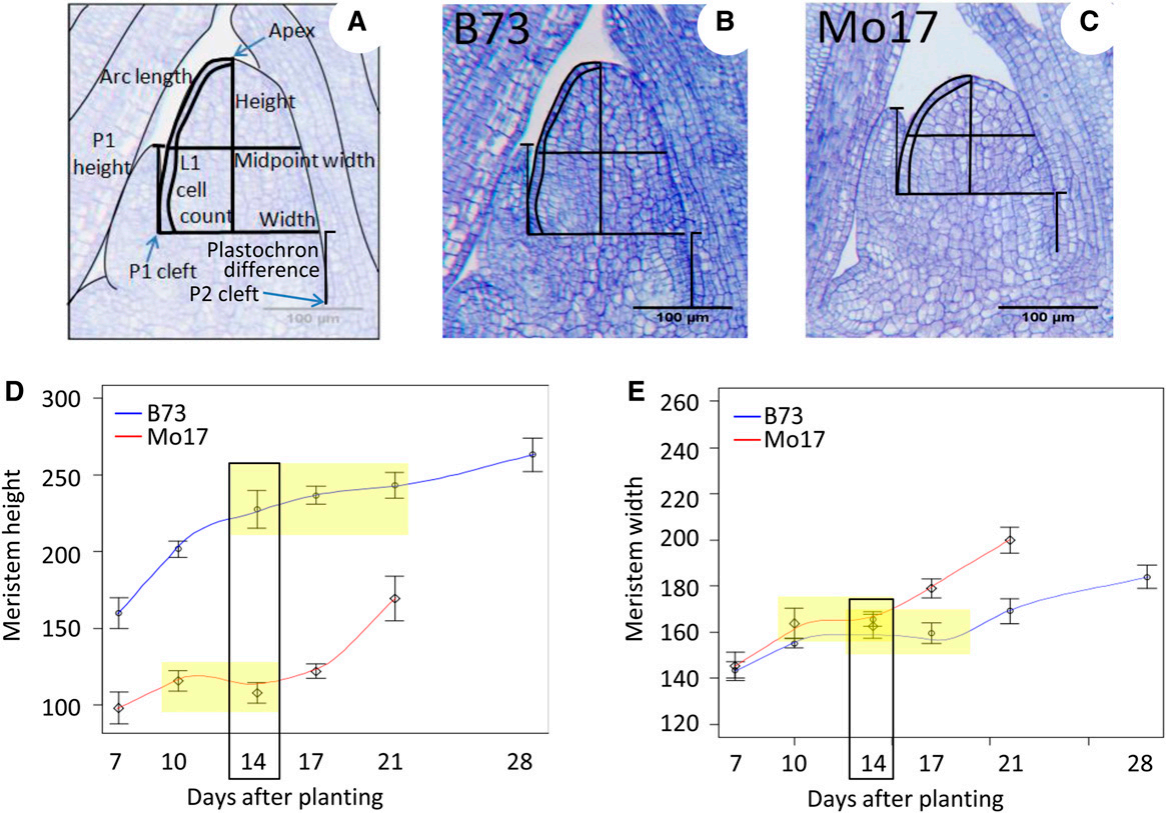
Shoot apical meristem (SAM) phenotypes and time points examined. Median longitudinal sections of the SAM in inbred lines B73 (B) and Mo17 (C), indicating measurements taken (A). B73 and Mo17 display markedly different meristem height but similar width. Changes in SAM height (D) and width (E) during vegetative development in B73 (blue) and Mo17 (red) 7−28 d after planting. Yellow boxes represent vegetative plateau; black box designates this overlap at 14 d. Mo17 transitions around 28 d after planting (not measured once transitioned). Error bars are weighted SE.

To assess the optimal time for assessing SAM architecture traits, B73 and Mo17 seed was planted in 6-inch square pots with five plants per pot in a 1:1 mix of black soil and SunGro potting soil, with the recommended application rate of 2 teaspoons per square foot of Osmocote Plus fertilizer. Plants were grown in a growth chamber with 16-hr days at 25° and 20° nights. Sixty B73 and 60 Mo17 individuals were grown for 4 wk with up to 10 plants sampled for histology at each time point (7, 10, 14, 17, 21, and 28 d after planting). An additional 40 of each genotype were grown in a second replication and sampled in a similar fashion at 7, 14, 21, and 28 d after planting. The results of these two experiments were combined by calculating the weighted mean and SE, allowing for the inclusion of the effect of the two separate experiments. Each genotype/week combination was represented by 9−20 measured images with the exception of Mo17 at 4 wk, where most of the SAMs had already transitioned and were not included. As a result of this study, 2 wk was used as the sampling time point for the remainder of the analyses because 2-wk-old SAMs had reached full vegetative size while not yet showing signs of transition (Figure 1, D−E, black and yellow boxes).

For the mapping and validation experiments, seeds were planted in 1-inch wide by 8-inch deep tubes placed in 10 × 20 racks using a 1:1 mix of black soil and either MetroMix or SunGro potting soil, with the recommended application rate of 2 teaspoons per square foot of Osmocote Plus fertilizer. Every third row of ten within flats were left empty to allow plants more room to grow, provide even air flow, and reduce edge effects, resulting in a total of 140 plants per rack. Plants were grown for 14 d in growth chambers with 16-hr d at 25° and 20° nights.

Eighty-six IBMRILs and the B73 and Mo17 parents were grown twice, each grow-out containing two replications of 10 plants per line, with lines randomized within each replication. All healthy plants were sampled for histology, and 15−39 images were measured per RIL, in addition to 83 B73 and 80 Mo17.

For the validation experiment, two B73-like (B034 and B063) and one Mo17-like (M049) NILs (Table S1) were selected to target the SAM_height_6 QTL on chromosome 5. These three lines as well as B73 and Mo17 were grown in 40 small blocks, each consisting of one plant per line and lines randomly distributed within each block. All normally growing plants were sampled for histology.

### Histology

For the IBMRIL population, shoots were dissected at 14 d, fixed in formalin-acetic acid-alcohol solution overnight, and embedded in paraffin according to Ruzin (1999). Serial, median longitudinal 8-μm sections were cut using a microtome, mounted on slides, stained with toluidine blue, and deparaffinized. A light microscope and Zeiss AxioVision software was used to capture SAM images.

For the time course study and NIL validation, shoots were dissected at 14 d and fixed in formalin-acetic acid-alcohol solution overnight. Tissues were then dehydrated and cleared using a series of ethanol and methyl salicylate according to Jackson and Hake (1999) before being stored in 100% methyl salicylate. Cleared tissue blocks were imaged directly on slides and captured as described previously.

### SAM architecture measurements

SAM images were measured for SAM width, height, arc length, midpoint width, P1 height, plastochron internode length, and arc cell count using ImageJ software (http://rsbweb.nih.gov/ij/). Width was measured from the point of insertion of the P1 leaf into the SAM, known as the P1 cleft (Figure 1A). Height was measured from the apex of the SAM to the width line, and arc length traced the outer distance from the apex to the P1 cleft. Midpoint width was defined as the width of the SAM at the midpoint of the height. P1 height was the distance from the P1 cleft to the tip of the P1. Plastochron internode length (PIL) was the vertical distance from the P2 to P1 cleft. Cells were counted in the L1 layer along the arc length. Average cell size (arc length divided by arc cell count), height/width ratio, and volume as a dome were also calculated as derived traits for each individual sample.

### Data analysis

Analysis of the raw phenotype data, including Pearson’s correlation coefficients, analyses of variance, and Student’s *t*-test (for comparing NILs to parental inbreds) were conducted in R (The R Project for Statistical Computing). Heritability was calculated as the additive genetic variance of the RIL (Va_RIL) divided by the total phenotypic variance according to Bernardo (2010).

The genetic map used for QTL mapping was derived from sequenced RNA in the IBMRIL (Li *et al.* 2013). QTL mapping was performed using QTLCartographer (NC State University) composite interval mapping (Model 6), with 10 background markers and 5-cM windows. To determine a significance threshold, 1000 permutations at α = 0.05 were conducted on each trait. All traits gave similar results, so the average logarithm (base 10) of odds (LOD) value of 3 was used as a cutoff to declare QTL significance. Confidence intervals were determined by a 1-LOD drop.

To map the correlation between gene expression in shoot apices and SAM phenotypes, Pearson’s correlation coefficients were calculated for each pairwise gene and trait combination across 86 IBMRILs. Significance thresholds were determined for each gene-trait combination individually at a comparison-wise threshold of *P* < 0.05 after 1000 permutations. Genes included in this analysis are only those expressed in some stage of the ontogeny of the B73 SAM (Takacs *et al.* 2012), as well as in shoot apices of the IBMRIL (Li *et al.* 2013).

## Results

### Growth of the maize SAM

To examine the architecture of the maize SAM throughout the vegetative phase of growth, we measured B73 and Mo17 at six time points from 1 to 4 wk after planting. Line means for time course data are available in Supporting Information, Table S2. Analysis of SAM width and to a lesser extent SAM height from the P1 cleft (Figure 1A) revealed a similar pattern of SAM development between the two inbreds, with a phase of decelerated growth rate surrounded by two periods of more rapid expansion (Figure 1, D and E). Thus, in addition to its role in organ initiation, the SAM seems to dynamically alter the rate of expansion of its stem cell pool throughout vegetative development. After the initial expansion of the SAM, this phase of delayed growth started around 14 d after planting for B73 and around 10 d for Mo17 (Figure 1, D and E, yellow boxes indicate leveling off of growth rate).

Another period of SAM expansion took place approximately 18−19 d for Mo17 and between 21 and 28 d (exact time point not known) for B73, or approximately 10 d before transition. During this time, the SAM again accumulated stem cells before it began to transition to an inflorescence meristem, as defined here by the presence of branch and spikelet pair meristems. Throughout this phase, the SAM rapidly became wider and taller (Figure 1, D and E), and its relative position in the plant began to shift upwards by 1−2 cm. At 28 d after planting, most (89%) Mo17 SAMs had reached transition, compared with 0% for B73.

Fourteen days after planting (black boxes in Figure 1, D and E) was selected as a common time at which to measure the vegetative size of the SAM in its phase of stem cell pool maintenance rather than proliferation, because both B73 and Mo17 SAMs had reached the plateau of full vegetative size at this age, but neither inbred at this time point had started to expand prior to transition.

### Quantitative variation for SAM architecture in maize

To determine the heritability of natural variation for a variety of SAM architecture traits, we examined B73 and Mo17 and a subset of 86 individuals of the IBMRIL population (Table S1). Measurements of 10 traits were initially used to characterize SAM architecture: height, arc length, width, and midpoint width from the P1 cleft; cell number in the L1 layer along the arc length; length of P1; PIL; and the derived traits of cell size, height.width ratio, and volume (Figure 1, A−C). The measurements of SAM traits in B73 and Mo17 were compared with the distributions observed in the RIL population to determine the type of segregation occurring for each trait (Table 1).

**Table 1:** Summary of SAM architecture traits in the IBMRIL population and parents

Meristem Trait	Parents		RILs		*P*-value*^a^*	Heritability
	B73	Mo17	Mean	Range		
Height, μm	188	104	133	103−178	<0.001	0.86
Arc length, μm	216	141	165	134−205	<0.001	0.82
Width, μm	138	142	135	116−155	<0.001	0.72
Midpoint width, μm	116	120	115	99−130	<0.001	0.71
Height of P1, μm	50	49	53	28−74	0.032	0.14
Cell count in arc, cells	17.0	12.3	13.9	11.1−17.8	<0.001	0.66
Volume, million μm^3^	5.10	1.50	2.40	1.41−4.39	<0.001	0.83
Height/width ratio	1.37	0.74	0.99	0.74−1.35	<0.001	0.88
Arc cell size, μm	13.2	11.7	12.2	10.2−14.5	NS	0.43
Plastochron internode, μm	70	67	68	56−84	<0.001	0.54

SAM, shoot apical meristem; IBMRIL, intermated B73 × Mo17 recombinant inbred line; RIL, recombinant inbred line.

a Significance of the effect of genotype within the RIL population.

**Figure 2:**
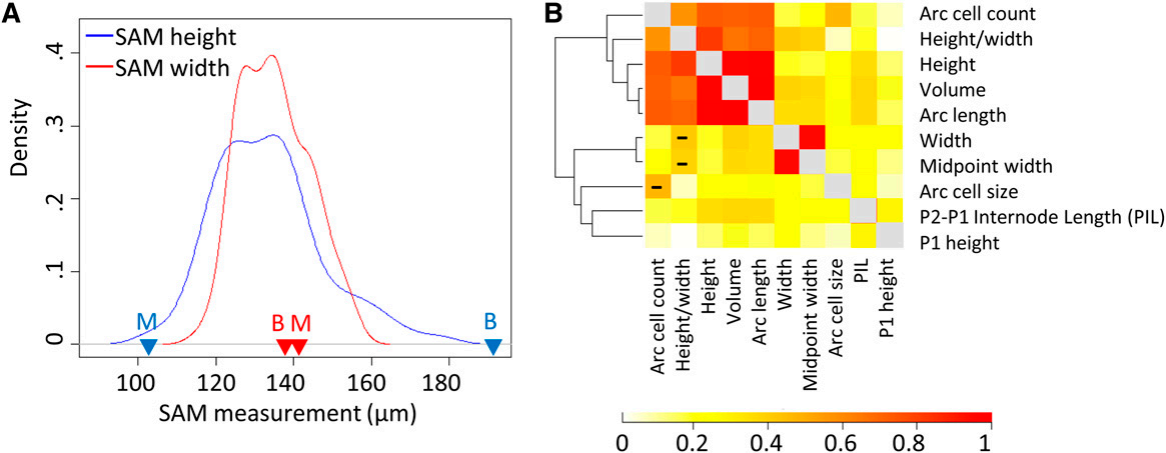
Distribution and relationship of traits. (A) Density distributions of shoot apical meristem (SAM) height (blue) and width (red) in the intermated B73 × Mo17 recombinant inbred line (IBMRIL) population. B73 and Mo17 are indicated as B and M, respectively. (B) Relationship of all SAM traits, both measured and derived, in the IBMRIL. SAM height, arc length, and volume are all highly correlated (r > 0.98), as are width and midpoint width (r > 0.94). Strengths of correlations are shown with absolute values; negative correlations are designated in the lower half of the matrix by “−”.

Distributions of the meristem traits followed two main patterns, exemplified by width and height (Figure 2A and Figure S1). For many SAM traits (height, height/width ratio, volume, arc cell count, and arc length), B73 and Mo17 exhibited substantially different values (*P* < 0.001; Table 1, Figure 2A, and Figure S1). The distributions of these traits in the IBMRIL showed a range of between-parent values and a positive skew, with more lines having values closer to the shorter SAM in Mo17. This could indicate that several alleles are needed in combination to create the extreme height observed in B73. Several other traits (SAM width, midpoint width, and PIL) displayed normal distributions (Figure 2A and Figure S1). The phenotype of B73 and Mo17 was quite similar for these traits, whereas the RILs showed substantial transgressive segregation (Table 1, Figure 2A, and Figure S1).

In addition to the extreme values of B73 and Mo17 observed for SAM height and related traits, these parental lines were also unique in their height/width ratio. Most RILs showed a height/width ratio very near 1 (average 0.99, Table 1). However, the B73 SAM was much taller than wide (ratio of 1.37, or 3.44 SD away from the mean), and Mo17 was wider than its height (ratio of 0.74; −2.34 SD). Only one Mo17-like IBMRIL showed a ratio insignificantly outside of these parental values.

Heritability estimates for SAM architecture were medium to high, ranging from 0.54 to 0.88 for significant traits (0.86 for height, 0.72 for width; Table 1). Several of the SAM architecture traits measured are likely to be under common genetic control mechanisms because correlations among the RILs (Figure 2B and Table S3) provided evidence for clusters of related traits. Some of these relationships reflected derived traits or related measurements. One group of correlated traits included height, arc length, volume, and height/width ratio, and the second group of correlated traits included width and mid-point width. These two groups showed high trait correlation within the groups and low correlation between them. All of the remaining three traits showed low correlation to the first two main phenotypic groups. PIL showed some correlation with SAM height, volume, and arc length but largely captured unique variation. SAM arc length was primarily caused by changes in cell number rather than size because SAM arc cell number was significantly different between the parents and across the population, correlated with other SAM height and arc length, and heritable.

Arc cell size and P1 height had the lowest heritability estimates and were not significant (at *P* < 0.01) among the RILs (Table 1). In the case of arc cell size, this may be attributable to deviation in cell size along the length of an individual SAM’s arc length or a lack of biological variation in the trait. In addition, it should be noted that relative arc cell size and count may likely differ from that of the meristematic cells in the central zone of the SAM. For P1 height, considerable growth occurs in a single primordium throughout the duration of any single plastochron, such that a P1 primordial early in the plastochron is much smaller than the same P1 leaf later in the plastochron, causing the trait to have substantial variation outside of genetic effect. Due to their low heritability these traits were not used for further analyses.

### Mapping QTL associated with SAM architecture

To identify regions of the genome associated with variation for SAM architecture, a QTL mapping analysis was conducted with all phenotypic measurements in the IBMRIL population (Table S4) combined with genetic marker data (7856 SNP markers; Li *et al.* 2013). Between six and nine QTLs were identified for every SAM trait, each contributing 4–23% of the trait variation (Table 2 and Table S5). The multiple regression model based on the combined significant QTL explained 55–74% of the total variation for each of the SAM traits (Table 2).

**Table 2:** Summary of SAM trait QTL

Meristem Trait	No. QTL (+)*^a^*	Range of PVE	Effect Range*^b^*	Model PVE
Height, μm	8 (6)	4–16%	3.02−6.1	0.68
Arc length, μm	8 (7)	5–9%	3.49−4.75	0.69
Width, μm	8 (4)	6–19%	2.38−4.87	0.61
Midpoint width, μm	8 (5)	7–12%	2.04−2.94	0.60
Cell count in arc, cells	9 (6)	4–23%	0.34−0.79	0.74
Volume, million μm^3^	7 (6)	5–16%	1.50−2.71E5	0.55
Height/width ratio	6 (4)	6–20%	0.24−0.57	0.55
Plastochron internode, μm	9 (5)	5–21%	1.47−4.23	0.66

SAM, shoot apical meristem; QTL, quantitative trait loci; PVE, phenotypic variation explained.

a Numbers in parenthesis indicate QTL with positive effect in B73.

b Effect ranges shown are absolute values.

Eight QTL were identified for SAM height, six of which were shared with arc length and/or volume QTL (Table 3). SAM height QTL were located on chromosomes 1, 2, 3, 5, and 6, with two each located on chromosomes 1 and 5, and the highest LOD and effect-size QTL located on chromosome 5. The SAM_height_6 QTL on chromosome 5 (Table 3) explained 16% of the variation in SAM height, and was also highly significant for all three related traits.

**Table 3:** QTL for SAM height

QTL	Chromosome	LOD	PVE	Effect*^a^*	cM Range*^b^*	Coincident SAM QTL
SAM_height_1	1	4.80	7%	4.06	717.91−729.41	
SAM_height_2	1	3.45	5%	3.62	1041.41−1052.91	Arc length, Plastochron internode
SAM_height_3	2	3.96	6%	3.74	301.21−310.91	Arc length
SAM_height_4	3	4.90	9%	−4.58	791.21−813.11	Arc length, volume
SAM_height_5	5	4.82	7%	4.33	772.71−781.71	Arc length, volume
SAM_height_6	5	9.25	16%	6.10	892.81−895.01	Arc length, volume
SAM_height_7	6	5.62	9%	4.49	303.01−317.71	Arc length, volume
SAM_height_8	6	3.03	4%	−3.02	489.71−499.31	

QTL, quantitative trait loci; SAM, shoot apical meristem; LOD, logarithm (base 10) of odds; PVE, phenotypic variation explained.

a Effect is substitution of Mo17 allele with B73.

b QTL intervals calculated by 1-LOD drop.

**Figure 3:**
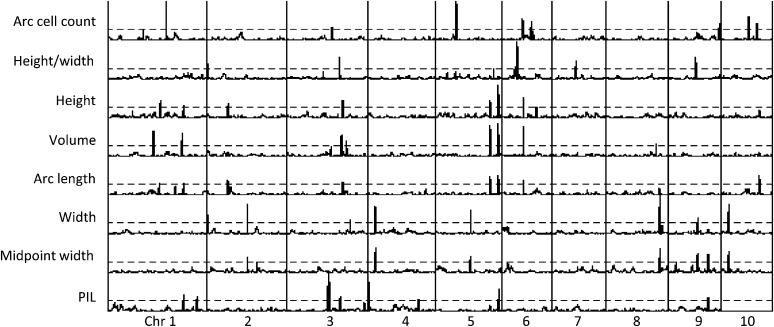
Mapping results across 10 chromosomes for the eight shoot apical meristem (SAM) architecture traits. The dashed line represents the significance threshold at logarithm (base 10) of odds (LOD) = 3, and LOD axis ranged from 0 to 14.3 for each trait. Height, volume, and arc length (all highly related traits) share many quantitative trait locus (QTL), whereas width and midpoint width share a different set of QTL. Arc cell count and plastochron timing have some QTL in common with other traits, but many are unique loci.

Eight QTL were mapped for SAM width, six of which were in common with midpoint width QTL (Figure 3 and Table S5). SAM width QTLs showed wider confidence intervals and were located across seven chromosomes (all but 1, 6, and 7). The most significant QTL for SAM width was shared by midpoint width and mapped to chromosome 8 and explained 19% of the variation.

The SAM architecture QTL regions were relatively well defined, with an average of 9.51 cM per QTL. Highly correlated SAM traits often showed QTL coincidence: QTL for SAM height were commonly found in the same locations as those for SAM arc length and volume, and SAM width and midwidth colocalized (Table S5 and Figure 3). Peaks significant for only a subset of correlated phenotypes frequently harbored smaller nonsignificant peaks for the correlated traits. None of the SAM height or height-related QTL was mapped to the same locations as SAM width or midwidth (Figure 3).

Traits that showed correlation among multiple different measures of SAM architecture reflected this in their QTL locations. For example, PIL was weakly correlated with other SAM traits but seemed to reflect some unique measures of SAM architecture. As predicted, five of nine QTL for PIL mapped to unique locations, whereas the other four coincided with height and/or arc length, volume, or midpoint width (Table S5).

The ability to map QTL for SAM architecture was independent of whether parental phenotypes showed distinct differences in the trait of interest (Table 1, Table 2, and Figure 2A), supporting the idea that QTL mapping can be successful in populations originating from similar parental phenotypes when transgressive segregation is present.

### Validation of a strong-effect SAM height QTL

**Figure 4:**
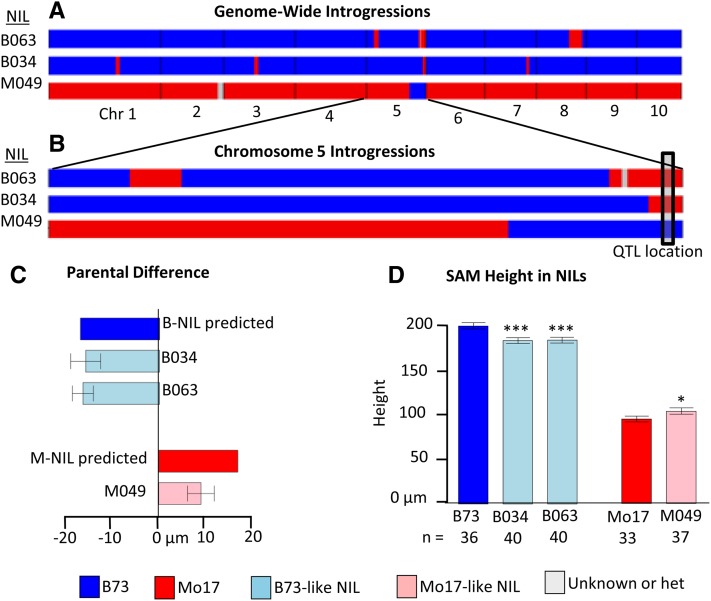
Validation of a quantitative trait locus (QTL) on chromosome 5 for shoot apical meristem (SAM) height. (A) Genome-wide view of near-isogenic lines (NILs) introgressions that are present in the measured NILs and (B) a close-up of chromosome 5 for the same lines, indicating the location of the QTL. (C) NILs containing the QTL region introgression show differences in SAM height approximately equal to the expected parental deviations based on estimated QTL effects for B73-like NILs, although slightly less than predicted for Mo17-like NILs. (D) All NILs were significantly different than their recurrent parent: *P < 0.05, ***P < 0.001 based on Student’s *t*-test.

We sought to validate the existence of SAM_height_6, a strong-effect QTL (16% variation explained, LOD 9.25) on chromosome 5 at 892.81−895 cM (Table 3 and Table S5) using a set of three NILs (Eichten *et al.* 2011). Two NILs (B034 and B063) had a largely B73 genome with regions of Mo17 introgression whereas the other line (M049) was mostly Mo17 with at least one introgressed region of B73. In each of these NILs, the introgression on chromosome 5 spanned the entire QTL confidence interval. Other introgressed regions (different for each NIL and not overlapping with other SAM height QTL) were present in the background, but these were minimal: B034, B063, and M049 contained 2.35%, 3.87%, and 2.56% total introgression, respectively. At least 33 individuals were measured for the parents and each of these three NIL genotypes. Line means for NILs examined are available in Table S6. The NIL genotypes were significantly different from the parents in the expected direction (Figure 4, C and D) based on the introgressed region (Figure 4, A and B). This analysis supports the existence of this SAM height QTL on chromosome 5 and suggests that it contributed 15% (16 of 105µm) of the variation in SAM height (Figure 4, C and D) based on B-like NILs. M049 showed a slightly smaller than predicted effect.

### Identifying potential candidate genes in QTL regions

**Figure 5:**
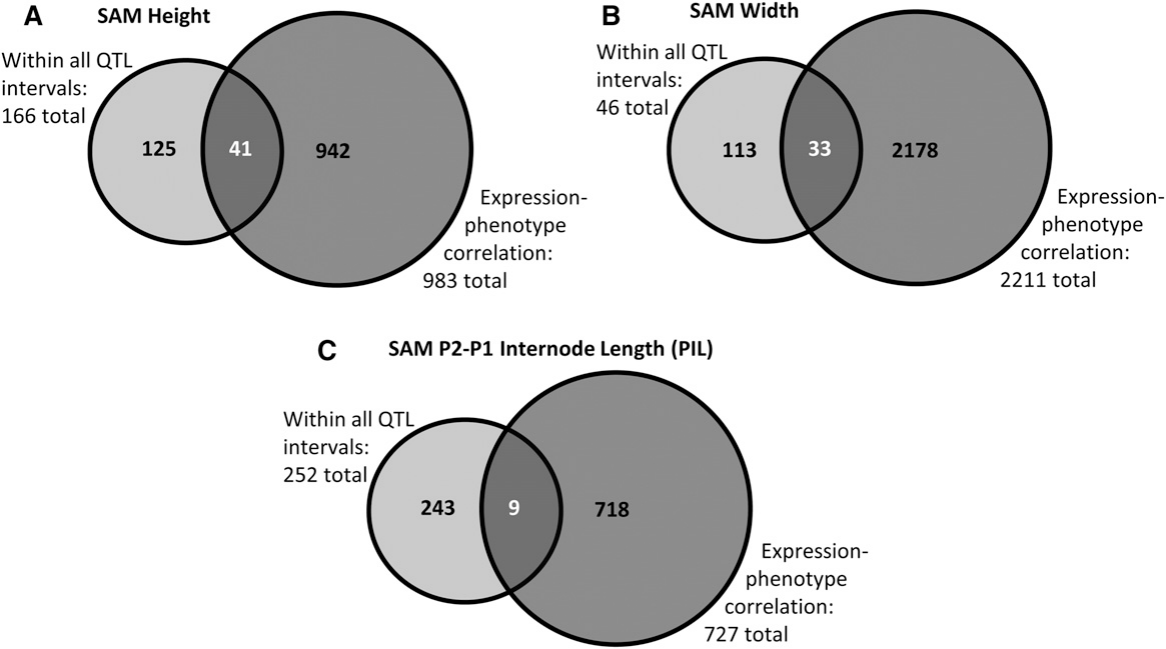
Identifying candidate genes based on expression. Numbers of genes expressed in the B73 shoot apical meristem (SAM) and intermated B73 × Mo17 recombinant inbred line (IBMRIL) apices located within the identified QTL intervals for each trait with expression significantly correlated (comparison-wise threshold *P* < 0.05) with SAM architecture for SAM height (A), width (B), and plastochron difference (C).

Three traits—SAM height, width, and PIL—representing the three different groups of correlated traits were further examined for potential candidate genes with the expression data. First, correlation analysis was conducted between gene expression in shoot apices (Li *et al.* 2013) and SAM architecture traits across the same set of IBMRILs to identify whether expression levels of some genes are correlated to SAM morphology differences and whether these genes are within genomic regions defined by the mapped QTL. There were 41, 33, and 9 cases for the three traits (Figure 5 A, B, and C, respectively) where significantly correlated gene expression coincided with genes located under QTL for that trait. These 83 genes include annotations related to sucrose and metal transport, homeobox genes, and growth-related factors (Table S7). These genes may be targets of future research in SAM growth and stem cell pool homeostasis, as well as potentially the control of maize vegetative development.

As these 83 genes would be expected to be expressed in the SAM and play a role in SAM formation or function, expression of these genes was examined across SAM ontogeny in B73 using expression data from Takacs *et al.* (2012). Levels and patterns of expression across SAM ontogeny varied greatly (Figure S2). Some genes were expressed in a stage-specific manner, whereas others showed increasing or decreasing expression levels throughout the development of the SAM. Using expression patterns may be one additional way to narrow down candidate genes for future studies targeting specific gene families and interactions.

## Discussion

### The maize SAM has three stages of development during vegetative plant growth

The morphology and growth rate of the maize SAM are not static. The most evident examples of dynamic growth are the changes that take place in the SAM before or at reproductive transition, among them the enlargement of the apex as the growth rate increases, a change in phyllotaxy and suppression of internodes in the flower, and an increased rate of initiation of primordia accompanied by decreased size of primordia at initiation (Lyndon and Battey 1985). In *Sinapis alba* these changes have been detected as early as 24 hr after long day floral induction; flower primordia initiated around 60 hr after (Bernier 1997). The dramatic enlargement of the SAM prior to initiation is thought to be due to an increase in the number of stem cells in the central part of the SAM to support the shift in phyllotaxy during flower formation (Ormenese *et al.* 2002).

In studying the dynamic growth of the SAM up until the point of reproductive transition (as defined here by the formation of spikelet pair meristems), we characterized the changes that took place during the vegetative growth of the plant. The time points analyzed indicated three distinct stages of vegetative SAM growth: an initial proliferation of the stem cell pool where SAM size increased, a maintenance phase where a balance was maintained between organ initiation and SAM maintenance, and finally a second expansion of the SAM in preparation for transition.

Previous research outlined in Chuck and Hake (2005) and Poethig (1990) indicate that the size of the shoot or even factors produced by the root system may be the important features in regulating the timing of reproductive development, in concert with the genetic pathways implicated in phase change. Sharman (1942a) too suggested that some minimum condition must be reached within the apex that tips a balance, causing the abrupt change from vegetative to reproductive growth. Perhaps part of that balance lies in the size of the SAM and the accumulation of an excess of cells in the meristematic pool that then triggers floral transition. This would explain the relative timeframe of the stages of SAM development of different inbreds during vegetative development, with earlier-flowering inbreds progressing faster through the stages of SAM growth (A. M. Thompson and G. J. Muehlbauer, unpublished data).

### Arc length of the tunica layer (L1) is influenced by cell number

A larger SAM may be caused either by an increase in cell number, cell size, or both. To comprehensively address which of these is the case in maize, the size and shape of cells in all regions of the SAM should be carefully examined. Because of the difficulty of obtaining accurate data for the more internal cells of the SAM, we investigated only cell number and size along the arc length in the L1 layer. Despite a wide range of SAM architecture present in the IBMRIL population and its parents, the size of these cells was not significantly different among genotypes (*P* > 0.05) and showed relatively low heritability (h^2^ = 0.43). Conversely, the number of cells present in this layer was found to be highly significant (*P* < 0.001) and slightly more heritable (h^2^ = 0.66; see Table 1). B73 and Mo17, the inbred parents of the population, exhibited very different morphologies as reflected by SAM height and arc length; this difference between the parents and throughout the population correlates with the increased number of cells but not with cell size. Thus, for the outermost layer of cells in the meristem, the differences observed in overall SAM size in the IBMRIL and its inbred parents were primarily related to the number of cells present, not cell size.

### SAM architecture is controlled by many QTL

Multiple QTL were identified for each of the traits analyzed. In a small population such as the subset used here, QTL analysis will tend to underestimate the number of loci contributing to the trait and overestimate the size of the effects (Beavis 1998). This finding indicates that the genetic control of maize SAM architecture may actually be controlled by even more QTL that were not identified. Similar results have been found for natural variation in other morphological characteristics, such as leaf shape in Antirrhinum (Langlade *et al.* 2005), tomato (Chitwood *et al.* 2013), and maize (Tian *et al.* 2011), as well as floral shape in Arabidopsis (Juenger *et al.* 2000). Also in maize, large numbers of small-effect additive QTL have been shown to be the basis of control of flowering time (Buckler *et al.* 2009), although larger-effect loci were identified for inflorescence traits (Brown *et al.* 2011).

### Genes controlling natural variation differ from those known to affect mutant morphologies

Many genes have been previously identified as affecting SAM size based on mutant phenotypes, including *Knotted-1* (Vollbrecht *et al.* 2000), *abphyl1* (Jackson and Hake 1999), *Extra cell layers1* (Kessler *et al.* 2006), *bladekiller1-R* (Woodward *et al.* 2010), *fasciated ear2* (*fea2*) (Taguchi-Shiobara *et al.* 2001), and *compact plant2* (Bommert *et al.* 2013). Several main processes also have been implicated in the initiation, function, or maintenance of the SAM, including the CLV-WUS feedback loop, KNOX, plant hormones, small RNAs, and chromatin structure and remodeling. However, genes known to be involved in these processes or directly impact the SAM did not coincide with the QTL we identified, which could indicate that these genes don’t play a significant role in the control of natural variation in maize SAM architecture, or perhaps that some of these genes do not show significant variation within the IBMRIL.

One of these genes, *fea2*, was implicated in a previous QTL study for kernel row number, a trait that correlated with inflorescence meristem size (Bommert *et al.* 2013). As mentioned, this gene was not identified as a QTL candidate for SAM size. In addition, expression of *fea2* was not correlated with SAM size across the RILs examined, and significant expression differences were not observed in shoot apex tissues between extreme groups in SAM size, even between B73 and Mo17. This finding supports the hypothesis that known SAM genes are simply not significantly different in the lines examined for their impact on the shoot meristem specifically.

Another reason for the lack of QTL coinciding with previously identified genes affecting SAM size is that mutations in these genes are often quite severe, affecting the expression of many downstream genes and having pleiotropic effects on plant growth and development. It may well be that downstream targets of these major genes are more likely to be implicated in controlling the range of natural variation observed in SAM architecture. Our results point to many basic physical structural functions on a cellular level: metal, sugar, and miscellaneous transport; cell organization and cell wall synthesis; lipid metabolism; and signaling. These pathways may be more likely to be responsible for the “fine-tuning” of the size and shape of the SAM.

Despite the lack of overlap with genes affecting SAM size, the 86 candidate genes identified did include annotations previously implicated in SAM function. Two classical maize genes (Schnable and Freeling 2011), *knotted interacting protein1* and *sucrose export defective1*, were among this subset, along with a gene annotated as a homeobox gene. Knotted-interacting and homeobox genes may play a role in shaping the maize SAM because genes in these families have been shown to be involved in SAM function (Jackson *et al.* 1994; Kerstetter *et al.* 1994; Hake *et al.* 1995; Belles-Boix *et al.* 2006) and alter leaf morphology and internode patterning (Nishimura *et al.* 2000; Smith and Hake 2003; Rosin *et al.* 2003). Several sucrose and metal transport annotations (as well as 16 other miscellaneous transport annotations) also were observed in the candidate subset; these classes of genes are differentially expressed in the apex cells of narrow sheath mutant plants (Zhang *et al.* 2007), which are paralogous to the WUSCHEL1-like homeobox transcription factors. An ethylene metabolism gene, three RNA transcription regulators, and two elongation factor protein genes also were among the potential candidates.

Interestingly, although several genes that encode transporters for auxin, cytokinin, and gibberellin have been shown to be involved in the formation and function of the SAM (Hay *et al.* 2002; Rupp *et al.* 2002; Scanlon *et al.* 2002; Kessler and Sinha 2004), these families were not identified in our analysis. Again, although it may be that these genes are not relevant to SAM architecture, an alternative explanation is that they simply are not differentially expressed in the IBMRIL population. In either case, our results support the presence of multiple undiscovered genes contributing to natural variation in meristem morphology that warrant further study.

## Supplementary Material

Supporting Information
