# Supplementary material for: Genetic Control of Maize Shoot Apical Meristem Architecture
Source: G3 (Bethesda). 2014 May 22;4(7):1327–37. doi: 10.1534/g3.114.011940 (PMC4455781; doi:10.1534/g3.114.011940)
Supplement: Supporting Information [file supp_4_7_1327__index.html]

Genetic Control of Maize Shoot Apical Meristem Architecture — Supporting Information 

# Genetic Control of Maize Shoot Apical Meristem Architecture

## Supporting Information for Thompson *et al.*, 2014

**Files in this Data Supplement:**

- Supporting Information - Figures S1-S2 and Tables S1-S7 (PDF, 232 KB)
- Figure S1 - Density distributions of remaining SAM traits in the IBMRIL population. (PDF, 108 KB)
- Figure S2 - Patterns of expression in candidate genes. (PDF, 112 KB)
- Table S1 - Genotypes used for IBM subset and NILs. (.xls, 44 KB)
- Table S2 - SAM measurements for time course experiment. (.xls, 23 KB)
- Table S3 - Correlation values for SAM traits. (.xls, 27 KB)
- Table S4 - SAM measurements for IBM RILs examined. (.xls, 33 KB)
- Table S5 - All QTL for SAM traits. (.xls, 35 KB)
- Table S6 - SAM data for NILs examined. (.xls, 23 KB)
- Table S7 - Annotations, locations, and RPM values of genes in Figure S2. (.xls, 54 KB)
