# Supplementary material for: Genetic Control of Maize Shoot Apical Meristem Architecture
Source: G3 (Bethesda). 2014 May 22;4(7):1327–37. doi: 10.1534/g3.114.011940 (PMC4455781; doi:10.1534/g3.114.011940)
Supplement: Supporting Information [file supp_g3.114.011940_011940SI.pdf]

G3· Genes | Genomes | Genetics

Genetic control of maize shoot apical meristem architecture

Addie M. Thompson[*], James Crants[*], Patrick S. Schnable[§], Jianming Yu[†], Marja C. P. Timmermans[‡], Nathan M. Springer[**], Michael J. Scanlon[§§], Gary J. Muehlbauer[*, **]

[*]Department of Agronomy and Plant Genetics, University of Minnesota, St. Paul, Minnesota 55108
[§]Department of Genetics, Development and Cell Biology, and Agronomy, Iowa State University, Ames, Iowa 50011
[†]Department of Agronomy, Iowa State University, Ames, Iowa 50011.
[‡]Cold Spring Harbor Laboratory, Cold Spring Harbor, New York 11724.
[**]Department of Plant Biology, University of Minnesota, Saint Paul, Minnesota 55108.
[§§]Department of Plant Biology, Cornell University, Ithaca, New York 14853.

Corresponding author:
Gary J. Muehlbauer
Plant Biology
Room 250 Biological Sciences
1445 Gortner Avenue
Saint Paul, MN 55108
612-624-2755
muehl003@umn.edu

DOI: 10.1534/g3.114.011940

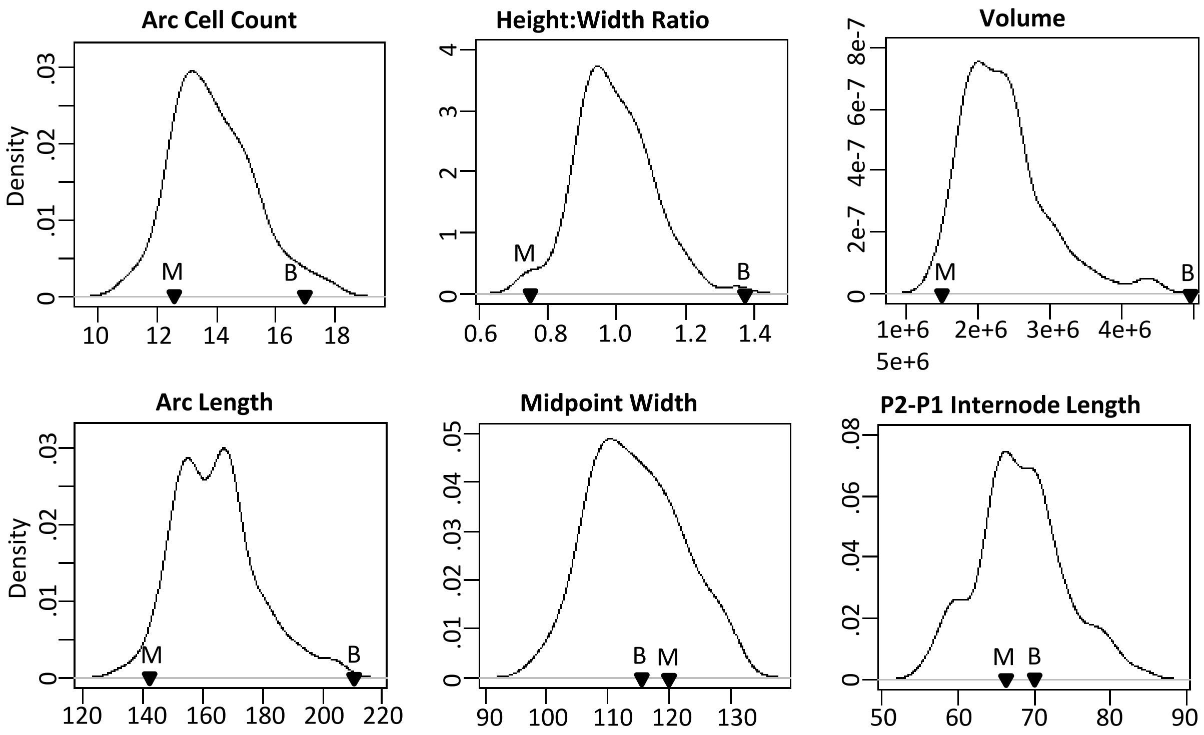

Figure S1 Density distributions of remaining SAM traits in the IBMRIL population. B73 and Mo17 are indicated as B and M, respectively.

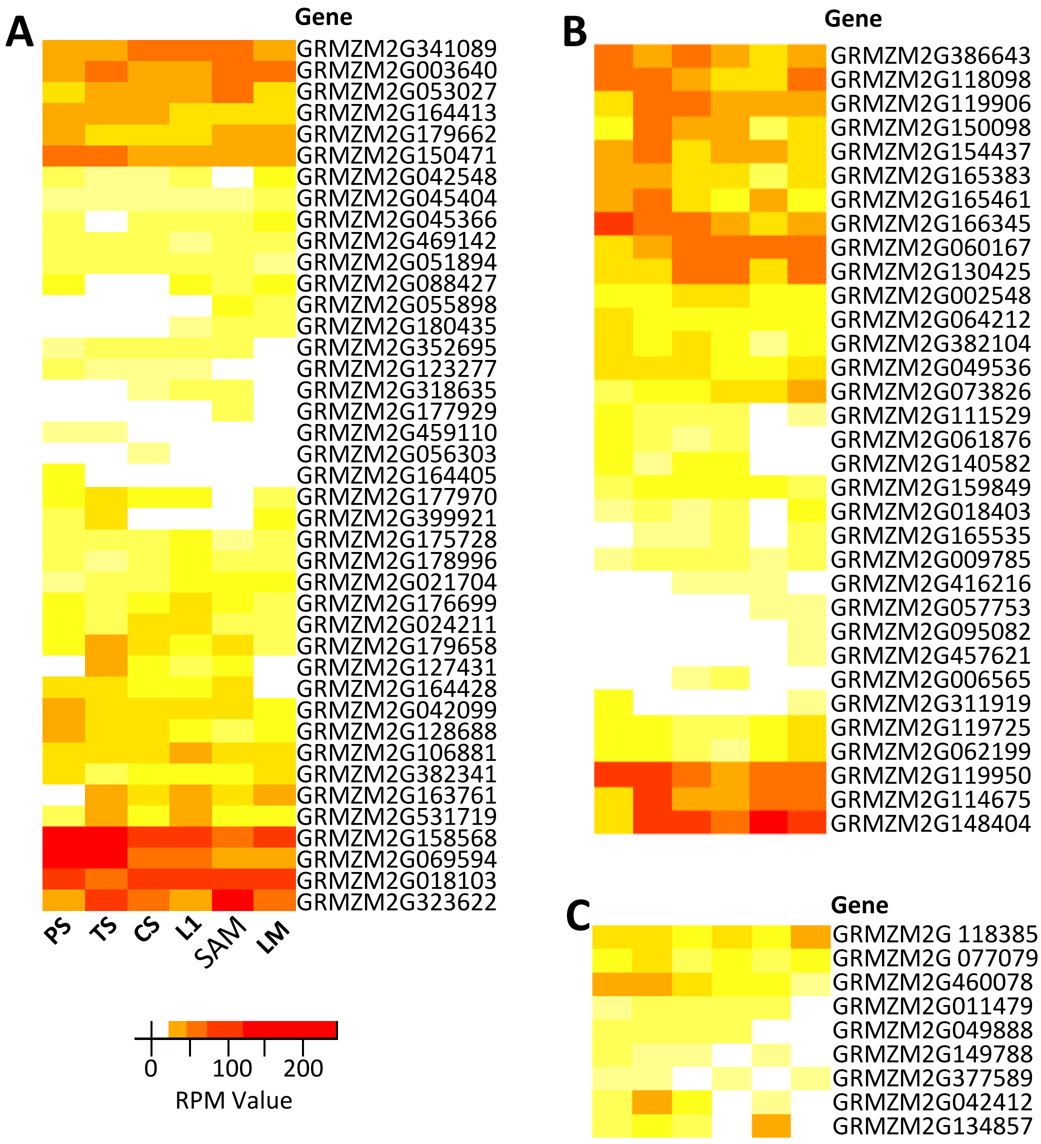

Figure S2 Patterns of expression in candidate genes. Gene expression across SAM ontogeny for genes expressed in the B73 SAM and IBMRIL apices located under QTL with expression significantly correlated with SAM architecture (see Figure 5) for SAM height (**A**), width (**B**), and PIL (**C**). PS=Proembryo stage SAM, TS=Transition stage SAM, CS=Coleoptile stage SAM, L1=L1 SAM of 14-day seedling, SAM=SAM of 14-day seedling, LM=Lateral Meristem of 14-day seedling. Annotations, locations, and RPM values of genes are listed in Table S4.

Tables S1-S7

Available for download at http://www.g3journal.org/lookup/suppl/doi:10.1534/g3.114.011940/-/DC1

Table S1 Genotypes used for IBM subset and NILs.

Table S2 SAM measurements for time course experiment

Table S3 Correlation values for SAM traits.

Table S4 SAM measurements for IBM RILs examined

Table S5 All QTL for SAM traits.

Table S6 SAM data for NILs examined

Table S7 Annotations, locations, and RPM values of genes in Figure S2.
