# Supplementary material for: Genetic Control of Maize Shoot Apical Meristem Architecture
Source: G3 (Bethesda). 2014 May 22;4(7):1327–37. doi: 10.1534/g3.114.011940 (PMC4455781; doi:10.1534/g3.114.011940)
Supplement: Supporting Information [file supp_g3.114.011940_FigureS1.pdf]

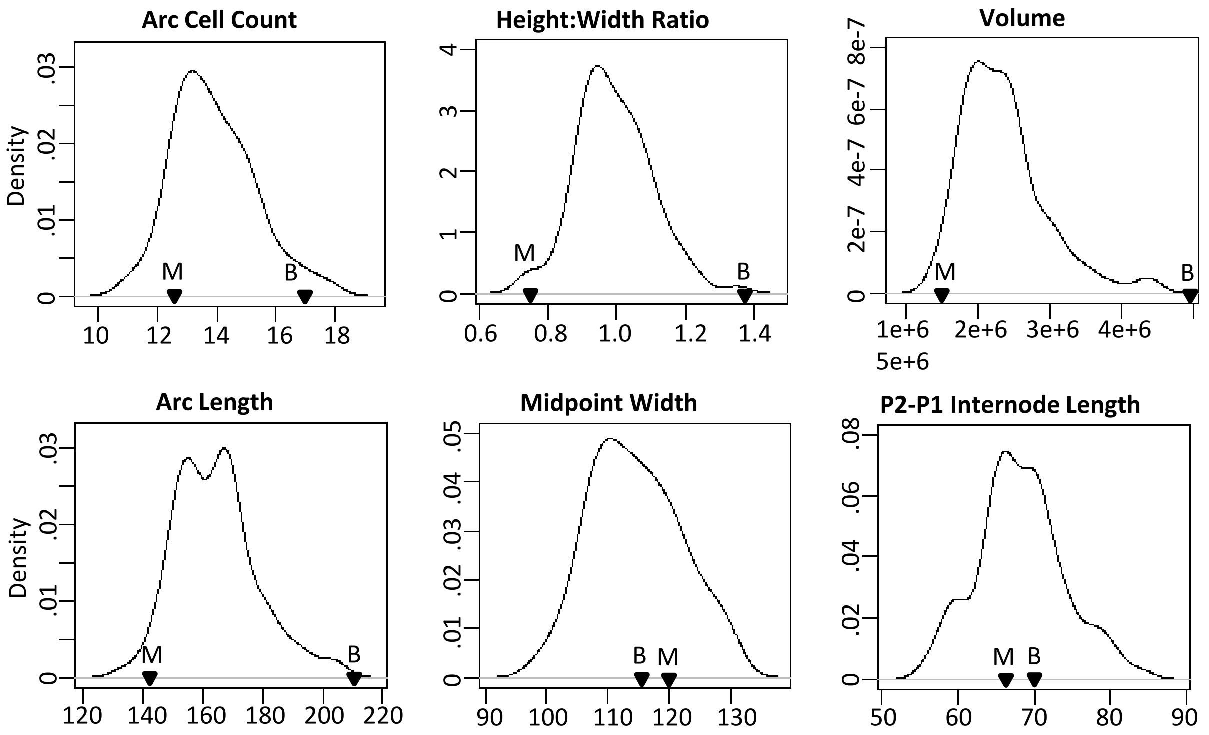

Figure S1 Density distributions of remaining SAM traits in the IBMRIL population. B73 and Mo17 are indicated as B and M, respectively.
