# Supplementary material for: Genetic Control of Maize Shoot Apical Meristem Architecture
Source: G3 (Bethesda). 2014 May 22;4(7):1327–37. doi: 10.1534/g3.114.011940 (PMC4455781; doi:10.1534/g3.114.011940)
Supplement: Supporting Information [file supp_g3.114.011940_FigureS2.pdf]

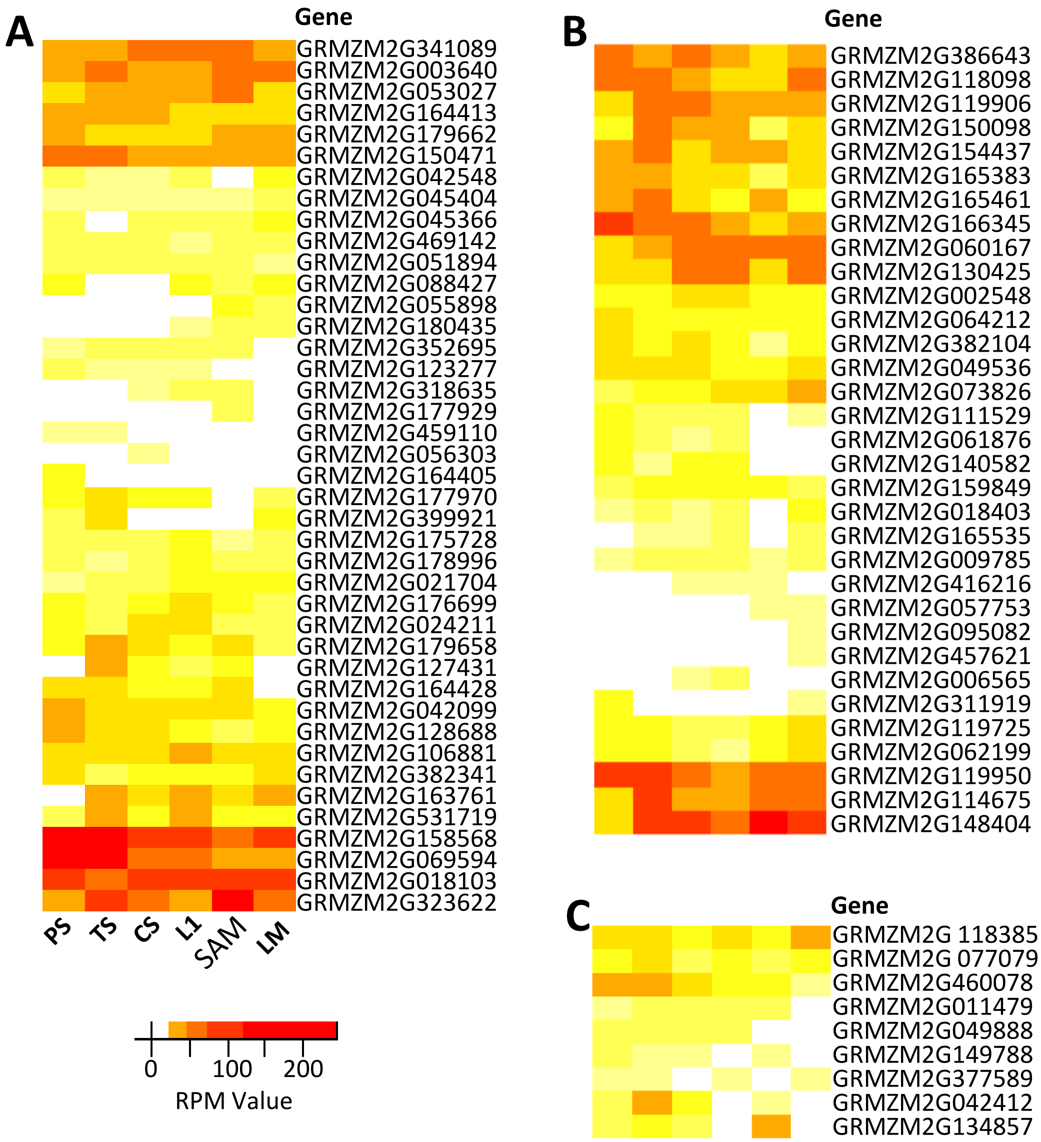

Figure S2 Patterns of expression in candidate genes. Gene expression across SAM ontogeny for genes expressed in the B73 SAM and IBMRIL apices located under QTL with expression significantly correlated with SAM architecture (see Figure 5) for SAM height (**A**), width (**B**), and PIL (**C**). PS=Proembryo stage SAM, TS=Transition stage SAM, CS=Coleoptile stage SAM, L1=L1 SAM of 14-day seedling, SAM=SAM of 14-day seedling, LM=Lateral Meristem of 14-day seedling. Annotations, locations, and RPM values of genes are listed in Table S4.
